# Is Burnout the Hidden Architecture of Academic Life in University Students? A Network Analysis of Psychological Functioning Within a Control–Value and Job Demands–Resources Framework

**DOI:** 10.3390/bs16040493

**Published:** 2026-03-26

**Authors:** Edgar Demeter, Dana Rad, Mușata Bocoș, Alina Roman, Anca Egerău, Sonia Ignat, Tiberiu Dughi, Dana Dughi, Alina Costin, Ovidiu Toderici, Gavril Rad, Radiana Marcu, Daniela Roman, Otilia Clipa, Roxana Chiș

**Affiliations:** 1 Center of Research Development and Innovation in Psychology, Faculty of Educational Sciences Psychology and Social Sciences, Aurel Vlaicu University of Arad, 310032 Arad, Romania; edgar.demeter@uav.ro (E.D.); alina.roman@uav.ro (A.R.); anca.egerau@uav.ro (A.E.); sonia.ignat@uav.ro (S.I.); dana.dughi@uav.ro (D.D.); alina.costin@uav.ro (A.C.); ovidiu.toderici@uav.ro (O.T.); gavril.rad@uav.ro (G.R.); radiana.marcu@uav.ro (R.M.); roxana.chis@uav.ro (R.C.); 2 Faculty of Psychology and Educational Sciences, Babeş-Bolyai University of Cluj-Napoca, 400029 Cluj-Napoca, Romania; musata.bocos@ubbcluj.ro; 3 Department of Psychology, Faculty of Socio-Humanistic Sciences, University of Oradea, 410087 Oradea, Romania; d.roman@uoradea.ro; 4 Department of Science of Education, Ștefan cel Mare University of Suceava, 720229 Suceava, Romania; otilia.clipa@usm.ro

**Keywords:** network analysis, academic burnout, academic motivation, self-regulated learning, academic engagement, generalized anxiety, self-esteem, instructional ratings, psychological networks

## Abstract

Academic functioning in university students emerges from the interplay of motivational, self-regulatory, emotional, and contextual processes. The present study examined the network structure linking academic motivation, self-regulated learning, academic engagement, academic burnout, generalized anxiety, self-esteem, and students’ ratings of instruction. Participants were 530 university students from Western Romania (Mage = 28.86, SD = 9.75; 87.5% women). Data were collected through an online cross-sectional survey using validated self-report instruments. A Gaussian Graphical Model was estimated using the EBICglasso procedure to examine the unique associations among the study variables and their relative structural importance within the network. The results indicated a moderately dense psychological network, with academic burnout emerging as the most structurally central node. Intrinsic motivation toward achievement, identified regulation, and performance control were positioned within the adaptive core of the network, whereas burnout, anxiety, amotivation, and low self-esteem clustered within the maladaptive region. Academic engagement occupied an intermediary position linking motivational and self-regulatory processes. Overall, the findings support a systems-oriented interpretation of academic functioning, suggesting that burnout represents a key convergence point in students’ psychological functioning, while self-determined motivation and self-regulated learning may serve as protective processes. These results highlight the value of network analysis for identifying psychologically meaningful intervention targets in higher education.

## 1. Introduction

Academic functioning in higher education is increasingly conceptualized as the result of dynamic interactions among motivation, self-regulated learning, engagement, emotional experiences, and wellbeing outcomes, rather than as the product of isolated psychological mechanisms. Self-Determination Theory (SDT) provides one of the most influential frameworks for understanding these interdependencies, proposing that students’ academic motivation varies in quality along a continuum from autonomous (intrinsic and identified) to controlled (introjected and external) regulation, with amotivation representing a state of disengagement or lack of intentionality (Deci & Ryan, 2008; Vallerand, 1997). Motivation that satisfies students’ basic psychological needs for autonomy, competence, and relatedness has been consistently associated with adaptive patterns of learning, persistence, and wellbeing (Niemiec & Ryan, 2009; Vansteenkiste et al., 2009; Ratelle et al., 2007). Conversely, controlled forms of motivation and amotivation tend to be linked to maladaptive outcomes, including emotional strain and academic withdrawal (Kusurkar et al., 2011; Elliot & Church, 1997).

Closely connected to motivational quality are students’ self-regulated learning (SRL) processes, which encompass forethought, strategic performance control, and self-reflection cycles that enable learners to plan, monitor, and evaluate their academic behavior (Zimmerman, 2002; Panadero, 2017). Theoretical models of SRL emphasize that effective regulation functions as a core mechanism through which motivation translates into sustained effort and academic engagement (Greene & Azevedo, 2007). Engagement itself is conceptualized as a multidimensional construct, comprising behavioral, emotional, and cognitive components that reflect students’ active investment in learning activities and their sense of belonging and participation in academic contexts (Fredricks et al., 2004; Skinner et al., 2008). Prior studies have shown that motivation, SRL, and engagement are jointly associated with academic achievement and positive adjustment, reinforcing the view that they operate as interconnected processes rather than discrete traits (Mega et al., 2014; Broadbent & Poon, 2015; Estévez et al., 2021).

At the same time, research grounded in the Study Demands–Resources and Conservation of Resources traditions has highlighted that students are also exposed to academic strain and risk of emotional exhaustion. Student burnout, typically characterized by exhaustion, cynicism, and reduced academic efficacy, has been linked to high study demands, insufficient resources, and reduced engagement (Alarcon et al., 2011; Lesener et al., 2020). Empirical work consistently indicates reciprocal associations between engagement and burnout, suggesting that these constructs form two poles within the same motivational–emotional system (Salmela-Aro et al., 2022; Jagodics & Szabó, 2023; Sun et al., 2024). Burnout has further been associated with impaired academic outcomes and increased likelihood of withdrawal intentions (Yusof et al., 2023), while structural-equation models show that emotional regulation and personal resources play an important mediating role in these associations (Tóth & Jagodics, 2025; Silva-Lorente et al., 2024).

Emotional functioning also plays a decisive role in students’ academic trajectories. Anxiety, particularly test and performance-related anxiety, has been shown to predict poorer academic outcomes and to co-occur with reduced engagement and self-efficacy (Cassady & Johnson, 2002; Soares & Woods, 2020; Woodrow, 2011). Meta-analytic evidence further indicates that anxiety relates negatively to performance, especially in high-stakes academic contexts (Huntley et al., 2019; Tang & He, 2023). In parallel, self-esteem has been identified as an important psychological resource in the academic domain, with prior research demonstrating its positive associations with achievement and adjustment (Rosenberg, 1965; Huang, 2011). More recent studies have shown that self-esteem and engagement jointly predict academic performance and perceived quality of life among university students (Acosta-Gonzaga, 2023; Vizoso et al., 2018; Garcia-Martinez et al., 2021).

Furthermore, students’ academic experiences are influenced not only by personal resources and emotional states but also by how they evaluate teaching and instructional environments. While the validity and interpretation of students’ ratings of instruction remain debated (Marsh & Roche, 1997; Clayson, 2009; Uttl et al., 2017), several studies suggest that perceived instructional quality is associated with students’ motivational orientations, engagement, and learning approaches (Diseth, 2007; Denson et al., 2010; Zabaleta, 2007). Such findings support the view that students’ evaluations of instruction are embedded in broader psychological processes rather than reflecting isolated judgments about teaching performance.

In addition to individual motivational and emotional resources, students’ academic functioning is shaped by broader contextual and interpersonal influences, including those stemming from the family environment. Although university students are often examined primarily in relation to institutional and individual determinants of adjustment, family-related dynamics may continue to play an important role in shaping academic wellbeing, stress, and engagement. Recent evidence shows that parental interference in adolescents’ educational and career-related choices is associated with greater depressive symptoms, partly through lower self-efficacy, higher school anxiety, lower engagement, and higher burnout (Angelini et al., 2026). Similarly, parenting styles and parental involvement have been linked to adolescents’ academic engagement and burnout, with more supportive and autonomy-promoting family environments generally associated with more adaptive academic functioning (Zhu et al., 2023). These findings reinforce the idea that students’ psychological functioning develops within a broader relational ecology, in which family expectations and involvement may either support motivational regulation and engagement or contribute to stress-related vulnerability.

Taken together, this body of literature portrays academic functioning as a multilayered psychological ecosystem, in which motivation, SRL, engagement, burnout, anxiety, self-esteem, and instructional perceptions are dynamically interrelated. However, most prior research has examined these constructs using regression or structural-equation models that specify directional and hierarchical relations between predictors and outcomes (Atik & Çelik, 2021; Zhen et al., 2017; Oriol-Granado et al., 2017). While these approaches have produced valuable insights, they implicitly assume that psychological variables influence outcomes in relatively linear and independent ways. Less is known about how these constructs operate when conceptualized as mutually interacting components of a single system, where influence may emerge from the configuration of relations rather than from individual predictors alone.

Recent methodological developments in educational psychology and network science have encouraged a shift beyond strictly latent or linear approaches toward models that conceptualize psychological variables as interdependent nodes within complex systems (Howard et al., 2021; Beachboard et al., 2011; Chiu & Chai, 2020). Within this framework, constructs such as motivation, self-regulated learning, burnout, anxiety, and engagement are not treated as isolated predictors or outcomes but as dynamically connected components of students’ academic functioning. This perspective allows for the identification of variables that may occupy different structural positions within the system, including central nodes, intermediary positions, or more peripheral indicators. As such, network analysis offers a more nuanced way of understanding how adaptive and maladaptive processes coexist, interact, and potentially reinforce one another in academic life. This systems-oriented approach is especially relevant in the study of academic functioning, where motivational, regulatory, and emotional variables may cluster together, transmit influence across domains, and reveal psychologically meaningful leverage points for intervention.

Building on these theoretical and methodological developments, the present study applies a network analytic approach to examine the interrelations among academic motivation (intrinsic, identified, controlled, and amotivation), self-regulated learning processes (forethought, performance control, self-reflection), academic engagement, academic burnout, generalized anxiety, self-esteem, and students’ ratings of instruction in a large sample of university students. Rather than treating these constructs as independent predictors of single outcomes, we conceptualize them as nodes in a psychological network whose structure may reveal emergent patterns of influence and clustering.

Accordingly, this study addresses the following research questions:

RQ1. How are motivation, self-regulated learning, engagement, burnout, anxiety, self-esteem, and instructional ratings interrelated when examined within a single psychological network?

RQ2. Which constructs emerge as central or structurally influential nodes within the network?

RQ3. Which constructs appear more peripheral within the network, and how do they cluster within adaptive versus maladaptive regions?

These research questions aim to clarify the relational structure of motivational, regulatory, emotional, and instructional variables in students’ academic lives and to advance a systems-oriented perspective on student wellbeing and academic adaptation.

## 2. Materials and Methods

The present study employed a cross-sectional survey design to examine the interrelations among academic motivation, self-regulated learning processes, engagement, burnout, anxiety, self-esteem, and students’ ratings of instruction within a university student population. Data were collected through an online questionnaire administered to students enrolled in higher education programs in Western Romania, using a convenience sampling approach. This regional context is relevant because it includes a heterogeneous higher-education population shaped by diverse educational and occupational trajectories, including both traditional-age students and adult learners returning to study. All participants completed a battery of validated psychological instruments measuring motivational regulation, academic engagement and burnout, generalized anxiety symptoms, self-regulatory learning strategies, self-esteem, and perceptions of instructional quality. The study was conducted in accordance with ethical research standards, participation was voluntary, and informed consent was obtained prior to survey completion. Descriptive statistics, bivariate correlations, and network analysis procedures were subsequently applied to explore the structure, centrality, and clustering patterns among the included variables.

### 2.1. Participants

The study employed a convenience sampling strategy and included 530 students from universities located in Western Romania who voluntarily participated in the online survey. Participants were recruited from multiple universities in the region and were analysed as a single pooled sample. Inclusion criteria required participants to be currently enrolled in a higher education program, to be aged 18 years or older, and to provide voluntary informed consent prior to participation. The sample was predominantly female (87.5%, n = 464), with 12.5% (n = 66) identifying as men. Participants’ ages ranged from 18 to 56 years, with a mean age of 28.86 years (SD = 9.75), based on 527 valid age responses. The sample should therefore be understood as a heterogeneous higher-education student sample rather than a strictly undergraduate cohort, as it included participants at different stages of educational attainment and academic progression. Additional demographic characteristics are presented in Table 1.

Before analysis, data completeness was examined for all study variables. Missingness was minimal, ranging from 0% to 0.57% across variables. Specifically, the age variable included 3 missing cases (0.57%; valid n = 527), while all variables included in the network model were complete. Because the network estimation used only the study variables included in the model, the final N for network estimation was 530. Listwise deletion was applied only for analyses involving variables with missing data.

The study was conducted in accordance with ethical research principles. Participation was voluntary, anonymity was ensured, and no identifying or sensitive personal data were collected. Prior to accessing the questionnaire, all participants were informed about the purpose of the study, the confidential treatment of their responses, and their right to withdraw at any time without consequences. Informed consent was obtained electronically from all participants before data collection.

### 2.2. Instruments

Academic Motivation Scale—College Version (AMS-C 28). Academic motivation was assessed using the Academic Motivation Scale—College Version (AMS-C 28; Vallerand et al., 1993), a 28-item measure grounded in self-determination theory and structured into seven 4-item subscales reflecting three intrinsic motivation dimensions, three extrinsic motivation dimensions, and amotivation. In line with the original instrument, items are rated on a 7-point Likert scale ranging from 1 (does not correspond at all) to 7 (corresponds exactly). The intrinsic motivation dimensions assessed motivation to know (α = 0.84), motivation toward achievement (α = 0.81), and motivation to experience stimulation (α = 0.82). The extrinsic motivation dimensions assessed identified regulation (α = 0.81), introjected regulation (α = 0.82), and external regulation (α = 0.78). The amotivation subscale, reflecting lack of purpose and psychological disengagement from learning, also showed good internal consistency (α = 0.86). In addition to the original source (Vallerand et al., 1993), the Romanian version has been psychometrically examined by Miulescu (2019), supporting its use in Romanian educational settings.

Generalized Anxiety Disorder Scale (GAD-7). Generalized anxiety symptoms were measured with the Generalized Anxiety Disorder Scale (GAD-7; Spitzer et al., 2006), a 7-item self-report instrument assessing worry, nervous tension, and core somatic manifestations of anxiety over the previous two weeks. Responses are recorded on a 4-point scale from 0 (not at all) to 3 (nearly every day). In the present sample, the scale demonstrated excellent internal consistency (α = 0.94). In addition to the original validation study (Spitzer et al., 2006), use of the Romanian version is supported by the psychometric evaluation reported by Cotiga et al. (2023).

Academic Engagement. Academic engagement was assessed using the engagement measure employed by S. Zhang et al. (2015), which conceptualizes engagement through the dimensions of vigor, dedication, and absorption. In the present study, the scale captured students’ energy, persistence, and cognitive-affective involvement in academic tasks and was scored on a 5-point Likert scale, with higher scores indicating stronger engagement. Internal consistency was excellent (α = 0.94). Because no Romanian validation study for this specific operationalization was identified, the scale was administered in translated form and interpreted with caution. This limitation is acknowledged in the manuscript.

Academic Burnout. Academic burnout was assessed using the burnout measure employed by S. Zhang et al. (2015), which operationalizes burnout through emotional exhaustion, detachment/cynicism toward study activities, and reduced academic efficacy. Items were rated on a 5-point Likert scale, with higher scores indicating greater burnout, and internal consistency in the present sample was high (α = 0.87). Although the scale is conceptually multidimensional, a composite burnout score was used in the network analysis because the focus of the study was on the overall structural role of burnout within the broader motivational–regulatory system rather than on the internal differentiation of burnout components. As with the engagement measure, no Romanian validation study for this specific version was identified; therefore, the measure was used in translated form and should be interpreted with appropriate caution.

Academic Self-Regulated Learning Questionnaire (ASLQ). Self-regulated learning was measured using the Academic Self-Regulated Learning Questionnaire (ASLQ; Nambiar et al., 2022), a 36-item instrument developed from Zimmerman’s cyclical model of self-regulated learning. The scale includes three dimensions: forethought and planning, performance control and monitoring, and self-reflection. In the original validation study, the 36 items were distributed across 10 forethought items, 19 performance-control items, and 7 self-reflection items. Items are rated on a 5-point Likert scale; to ensure consistency with the original validation, the anchors should be reported as 1 (strongly disagree) to 5 (strongly agree). In the present sample, the overall internal consistency of the ASLQ was excellent (α = 0.94). To our knowledge, a Romanian psychometric validation of the ASLQ has not yet been published. Accordingly, the scale was used in translated form, and this lack of local validation is acknowledged as a limitation.

Rosenberg Self-Esteem Scale (RSES). Global self-esteem was assessed using the Rosenberg Self-Esteem Scale (Rosenberg, 1965), a 10-item instrument measuring global self-worth. Items are rated on a 4-point Likert scale, with higher scores indicating higher self-esteem after reverse-coding the negatively worded items. In the present sample, reliability was good (α = 0.86). In addition to the original source (Rosenberg, 1965), the use of the scale in Romanian populations is supported by the psychometric study reported by Robu et al. (2015).

Universal Student Ratings of Instruction (USRI). Perceptions of instructional quality were assessed using the Universal Student Ratings of Instruction scale (USRI; Beran & Violato, 2009), which captures students’ evaluations of instructional clarity, course organization, feedback quality, fairness of assessment, and perceived learning outcomes. Responses were aggregated into a global indicator of instructional quality, and internal consistency in the present sample was excellent (α = 0.97). Although the instrument has strong support in its original context, we did not identify a dedicated Romanian validation study for the USRI. Therefore, the scale was used in translated form and interpreted cautiously, and this issue is acknowledged in the limitations section.

### 2.3. Data Analysis

All statistical analyses were conducted using SPSS (version 29) and JASP (version 0.18). Descriptive statistics (means, standard deviations, ranges, and distribution indicators) were computed for all study variables to characterize the sample and provide an initial overview of the central tendency of and variability in the motivational, regulatory, engagement, burnout, self-esteem, instructional perception, and anxiety indicators. Scale reliability was evaluated prior to the main analyses, and the corresponding internal consistency values are reported in the Instruments section.

Bivariate associations among the variables were first explored using Pearson correlation coefficients in order to provide a transparent descriptive overview of the observed pairwise relations among motivational orientations, self-regulated learning processes, academic engagement, burnout, self-esteem, students’ perceptions of instruction, and generalized anxiety. These correlations were examined descriptively and visualized through a heatmap, but they were not used to determine the network structure itself. The network model was estimated separately using regularized partial correlations, which capture unique associations between nodes after controlling for all remaining variables in the system.

To examine the multivariate organization of the psychological system formed by these constructs, a psychological network model was devised. A Gaussian Graphical Model (GGM) was computed using the EBICglasso estimator, which applies graphical least absolute shrinkage and selection in order to obtain a sparse partial correlation network and reduce spurious associations. Model selection was guided by the extended Bayesian information criterion (EBIC) with γ = 0.50. All variables were treated as continuous, and edges are interpreted as regularized partial correlations representing unique associations between nodes after controlling for the rest of the network.

**Figure 1:**
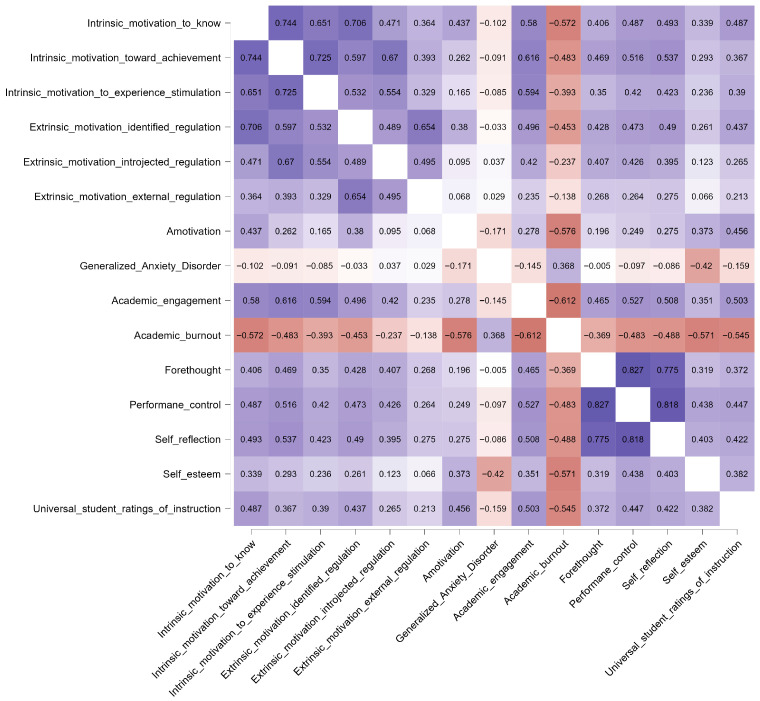
Correlation heatmap of study variables based on Pearson correlation coefficients. Note: Color intensity reflects the magnitude of the correlations, with darker shades indicating stronger associations and lighter shades indicating weaker ones. The numerical values in each cell represent the Pearson correlation coefficients, allowing the identification of both positive and negative relationships.

**Figure 2:**
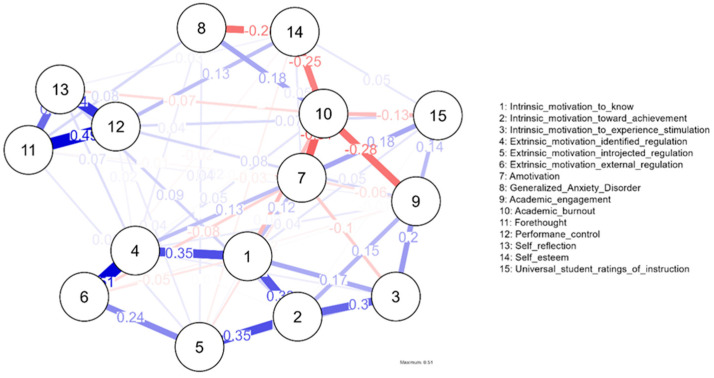
Network plot.

**Figure 3:**
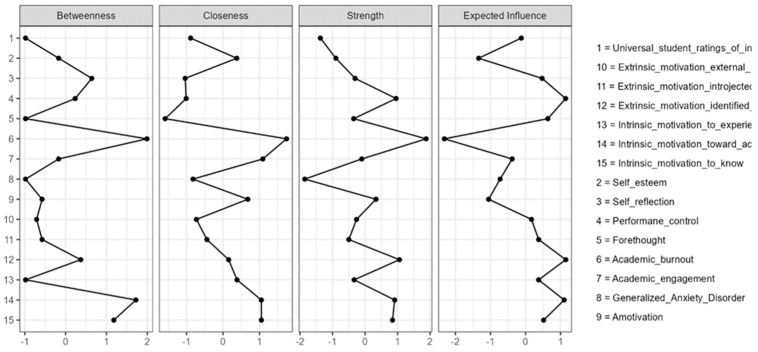
Centrality plot.

Network structure was evaluated using four standard centrality indices: strength, closeness, betweenness, and expected influence. Strength reflects the overall magnitude of a node’s direct connections with the rest of the network. Closeness indicates how near a node is to all other nodes in the network based on the shortest indirect paths, and is therefore interpreted as an indicator of how efficiently a node is embedded within the broader structure. Betweenness reflects how often a node lies on the shortest path between other nodes and is commonly interpreted as an index of potential intermediary or bridging position. Expected influence is similar to strength but retains the sign of the edges, indicating whether a node is connected predominantly through positive or negative associations. For comparability and visualization purposes, the centrality values reported in Table 2 and illustrated in Figure 1, Figure 2 and Figure 3 are standardized scores (z-scores), rather than raw index values. Accordingly, positive values indicate that a node scores above the network mean on a given index, whereas negative values indicate that it falls below the mean.

In addition, local clustering was examined using four complementary weighted clustering coefficients (Barrat, Onnela, WS, and Zhang). As with the centrality metrics, the clustering values reported in Table 3 are standardized scores (z-scores) derived for comparative interpretation across nodes. Therefore, negative values should not be interpreted as impossible raw coefficients but rather as indicating below-average local clustering relative to the other nodes in the network.

The stability and accuracy of the estimated network were assessed using nonparametric bootstrapping procedures implemented using the bootnet package, including confidence intervals for edge weights and correlation stability (CS) coefficients for centrality indices.

## 3. Results

Descriptive characteristics of the sample are presented in Table 1. Overall, the sample showed relatively high levels of intrinsic and identified motivation, academic engagement, self-reflection, self-esteem, and positive ratings of instruction, whereas burnout was lower on average. The corrected AMS-C 28 values are consistent with the 7-point response format of the instrument.

To provide a transparent overview of the bivariate associations among the study variables, a correlation heatmap based on Pearson correlation coefficients was generated (Figure 1). The heatmap visually summarizes the direction and magnitude of the pairwise associations among the study variables. The full Pearson coefficients are embedded in the heatmap cells.

The Pearson correlation heatmap (Figure 1) provides a descriptive overview of the pairwise associations among the study variables. In summary, motivational and self-regulated learning variables were positively interrelated and were generally positively associated with engagement, self-esteem, and instructional ratings, whereas burnout and anxiety showed the opposite pattern. Full Pearson coefficients are displayed in Figure 1.

Centrality indices were inspected using standardized estimates (Table 2) in order to identify the most structurally influential and functionally relevant nodes in the network. To further characterize the local organization of the psychological system, standardized clustering coefficients were examined using four complementary indices (Table 3).

**Figure 4:**
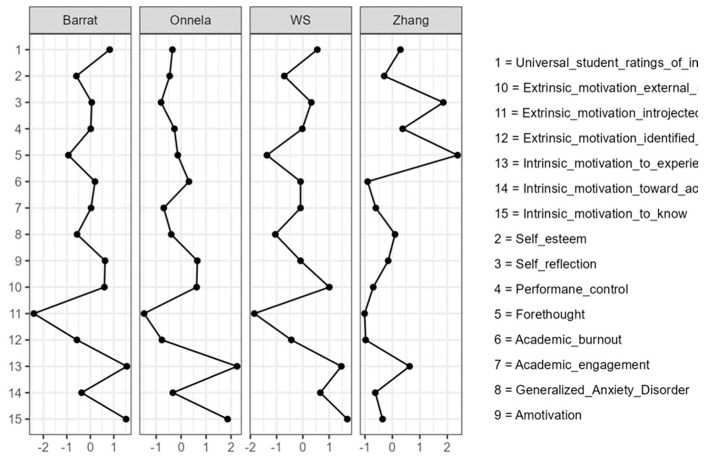
Clustering plot.

The estimated psychological network consisted of 15 nodes and 63 non-zero edges out of 105 possible pairwise connections, resulting in a sparsity value of 0.40. This indicates a moderately dense network in which a substantial proportion of variables remained uniquely connected after regularization. Bootstrap analyses indicated acceptable stability of the estimated network and centrality indices. The main centrality, clustering, and edge-weight results are summarized in Table 2, Table 3 and Table 4 and Figure 2, Figure 3 and Figure 4.

As shown in Table 2 and Figure 3, academic burnout occupied the most central position in the network across several indices. Intrinsic motivation toward achievement, identified regulation, and performance control also showed relatively prominent positions, whereas generalized anxiety, self-esteem, and students’ ratings of instruction were less central overall. Academic engagement showed a mixed profile, with stronger relative proximity within the network than direct connectedness.

To further characterize the local organization of the psychological system, clustering coefficients were examined using four complementary indices (Barrat, Onnela, WS, and Zhang). These indices indicate the extent to which each node forms cohesive local neighborhoods with its adjacent nodes, thereby reflecting whether psychological processes operate in locally integrated sub-systems or as more diffuse, weakly connected structures (Table 3).

Table 3 and Figure 4 summarize the local clustering profiles across nodes. Intrinsic motivation to know and intrinsic motivation to experience stimulation showed the highest relative clustering across several indices, whereas identified regulation and introjected regulation displayed lower clustering overall. The self-regulated learning variables, forethought, performance control, and self-reflection, showed mixed clustering profiles across indices. In contrast, academic burnout, generalized anxiety, academic engagement, and self-esteem generally displayed lower or less consistent local clustering, suggesting less cohesive local neighborhoods relative to the more tightly grouped motivational nodes.

The edge-weight matrix was examined in order to identify the strongest unique associations in the network and to clarify the functional pathways linking motivational, regulatory, emotional, and outcome variables. Edge weights represent regularized partial correlations, meaning that each connection reflects the unique association between two nodes after controlling for all remaining variables in the network (Table 4).

The strongest edge weights are reported in Table 4. The largest positive connections were observed among motivational and self-regulated learning variables, whereas the largest negative connections involved academic burnout, anxiety, engagement, and self-esteem. These patterns are visually reflected in the network plot shown in Figure 2.

Figure 2 presents the estimated EBICglasso network. Positive edges are shown in blue and negative edges in red, with thicker lines indicating stronger regularized partial correlations.

Figure 3 displays the standardized centrality estimates for all nodes.

Figure 4 displays the standardized clustering coefficients across nodes.

Overall, the descriptive, correlational, and network analyses showed a structured pattern of associations among motivational, self-regulatory, emotional, and instructional variables. Academic burnout showed the highest relative centrality values, whereas intrinsic motivation toward achievement, identified regulation, and performance control also occupied relatively prominent positions in the network. The strongest positive and negative edge weights, together with the clustering estimates, are reported in Table 2, Table 3 and Table 4 and Figure 2, Figure 3 and Figure 4. Their theoretical implications are considered in the Section 4.

## 4. Discussion

The present study used a psychological network approach to examine how different forms of academic motivation, self-regulated learning, engagement, burnout, generalized anxiety, self-esteem, and students’ ratings of instruction are conditionally associated within the same psychological system. The estimated network contained 15 nodes and 63 non-zero edges out of 105 possible, yielding a sparsity of 0.40 and indicating a relatively dense structure in which variables were substantially interconnected rather than behaving as isolated predictors or outcomes. This pattern is consistent with contemporary perspectives that conceptualize academic functioning as a transactional and multicomponent system in which motivational, regulatory, emotional, and instructional processes are closely related (Schunk & Zimmerman, 2012; Zimmerman, 2002). At the same time, because the present design is cross-sectional, the network should be interpreted as representing conditional associations rather than temporal or causal pathways.

Across all centrality indices, academic burnout emerged as the most central node in the network, with the highest betweenness, closeness, and strength, together with the most negative expected influence. This pattern suggests that burnout occupies a structurally prominent position linking motivational, self-regulatory, and emotional variables within the estimated configuration. In the present network, burnout was negatively connected with engagement, intrinsic and identified motivation, self-esteem, and students’ ratings of instruction, and positively connected with amotivation and generalized anxiety.

These results are compatible with the Job Demands–Resources (JD–R) model, which conceptualizes burnout as a key manifestation of prolonged imbalance between demands and available resources (Bakker & Demerouti, 2007). In the present network, variables such as intrinsic and identified motivation, self-regulated learning, and self-esteem were more closely aligned with the adaptive region of the network, whereas burnout occupied a relatively central position within the strain-related region. The strong negative edges between burnout and engagement, and between burnout and self-esteem, are consistent with prior findings showing that student burnout is associated with lower engagement, poorer academic outcomes, and reduced wellbeing (Schaufeli et al., 2002; Salmela-Aro & Upadyaya, 2014; Widlund et al., 2021; Cong et al., 2024; Olson et al., 2025). From the perspective of Conservation of Resources theory, the negative expected influence of burnout is compatible with a configuration in which burnout is linked to multiple unfavorable associations across the network, although temporal propagation cannot be inferred from the present cross-sectional design (Hobfoll, 1989).

The finding that burnout, rather than generalized anxiety, occupied the most central position in the academic network resonates with recent studies that place burnout at the core of student distress and performance difficulties (S. Zhang et al., 2015; Schaufeli et al., 2002). The present network adds a structural perspective to this literature by showing that burnout displayed higher relative centrality than anxiety when both were modelled simultaneously alongside motivational and self-regulatory variables.

On the adaptive side of the network, intrinsic motivation toward achievement and extrinsic motivation via identified regulation emerged as key hubs. Both showed high strength and positive expected influence, and intrinsic achievement motivation also displayed high betweenness, indicating that these constructs occupy central positions within the motivational–regulatory architecture.

These findings dovetail with Self-Determination Theory (Deci & Ryan, 2000; Vallerand et al., 1992, 1993) and with work on self-determined motivational profiles among students (Litalien et al., 2019; Guay et al., 2008; Liu et al., 2009). Intrinsic motivation oriented toward mastery and accomplishment, as measured via the Academic Motivation Scale (Vallerand et al., 1993), appears to occupy a central adaptive position that is consistent with higher engagement and closer links with self-regulated learning processes.

The strong connections between intrinsic and identified motivation and the self-regulated learning subscales (forethought, performance control, self-reflection), measured with the ASLQ (Nambiar et al., 2022), support the idea that self-determined forms of motivation are more conducive to strategic, future-oriented learning and metacognitive monitoring (Schunk & Zimmerman, 2012; Zimmerman, 2002). The network thus suggests a motivational–regulatory configuration in which intrinsic achievement motivation and identified regulation are closely connected with SRL processes, academic engagement, and more positive student evaluations of teaching (Beran & Violato, 2009).

Performance control and forethought were among the most influential self-regulation nodes, with high strength and positive expected influence. Together with self-reflection, they formed a densely interconnected triad, indicating a tightly integrated self-regulatory module. This is consistent with Zimmerman’s cyclical model of self-regulated learning, which highlights forethought (planning and goal-setting), performance control (strategy use and self-monitoring), and self-reflection as core phases of SRL (Zimmerman, 2002; Schunk & Zimmerman, 2012).

In the network, these SRL processes occupied intermediate positions between motivational nodes and engagement, suggesting that they may represent important connecting processes within the broader configuration. More specifically, self-determined motivation was closely linked to engagement and lower burnout, while self-regulated learning variables were positioned in ways consistent with a linking or intermediary role. This pattern is broadly consistent with prior literature linking self-regulation and self-efficacy to more adaptive academic and emotional functioning (Guay et al., 2003; Wang et al., 2022; S. Zhang et al., 2015), although the present study does not address temporal ordering.

Generalized anxiety symptoms showed negative strength and expected influence and a less central position compared with burnout. Amotivation also displayed a markedly negative expected influence. Structurally, both variables appeared more closely linked to burnout, low self-esteem, and low engagement than to the more central motivational and self-regulatory nodes. This pattern is consistent with a configuration in which anxiety and amotivation cluster within the more maladaptive region of the network, although no temporal ordering can be inferred.

This configuration is compatible with Control–Value Theory (Pekrun, 2006), which posits that achievement emotions, including anxiety, arise from appraisals of perceived control and value in academic tasks. Our network suggests that lower motivational and self-regulatory resources, together with higher burnout, were associated with higher levels of anxiety within the network configuration (Pekrun et al., 2017; Putwain et al., 2018). The strong negative edge between anxiety and self-esteem also fits with classic work on self-evaluation and psychological distress (Rosenberg, 1965).

Similarly, the configuration of amotivation, linked to burnout and reduced engagement, echoes prior work showing that amotivation is associated with negative adjustment and disengagement from school (Vallerand et al., 1992; Güngör & Sari, 2022). The network structure suggests a maladaptive cluster in which amotivation, burnout, anxiety, and reduced self-esteem tend to co-occur within the same region of the network.

Beyond individual paths, the present study contributes methodologically by applying regularized partial correlation networks to a broad set of academic variables. Network analysis is increasingly used in psychopathology research to conceptualize disorders as systems of mutually interacting symptoms (Borsboom, 2017; Fried & Cramer, 2017; Fried et al., 2016) and, more recently, in educational contexts to model relations among burnout, sleep quality, and internalizing symptoms (Chen et al., 2025; X. Zhang et al., 2025). Our findings extend this approach to the domain of academic motivation and SRL, showing that a network lens can reveal which constructs behave as hubs, bridges, or endpoints within a complex academic ecosystem.

Methodologically, the use of EBICglasso and regularized partial correlation networks (Epskamp & Fried, 2018) allowed us to move beyond traditional regression and SEM models that treat variables as independent predictors of single outcomes. Instead, we identified an emergent architecture that integrates motivation, self-regulation, engagement, burnout, and anxiety into a single system. This aligns with calls for multivariate network approaches in psychological science (Borsboom et al., 2021) and adds to existing evidence that burnout and engagement are intertwined yet distinguishable states within the demands–resources framework (Schaufeli et al., 2002; Salmela-Aro & Upadyaya, 2014; Widlund et al., 2021; S. Zhang et al., 2015).

From an applied perspective, the network suggests that interventions targeting burnout, intrinsic/identified motivation, and self-regulated learning may yield the largest systemic benefits. Reducing study-related demands and bolstering resources such as autonomy support, competence feedback and meaningful goals, core components of SDT and JD–R–consistent interventions (Deci & Ryan, 2000; Bakker & Demerouti, 2007; Guay et al., 2008), may weaken the central maladaptive node (burnout) while strengthening adaptive hubs. At the same time, training in SRL strategies (forethought, performance control, reflective monitoring) may strengthen the self-regulatory processes that occupied relatively prominent and intermediary positions in the network (Zimmerman, 2002; Schunk & Zimmerman, 2012; Nambiar et al., 2022).

The positive links between adaptive nodes and students’ ratings of instruction also highlight that instructional quality is embedded within the same system: teaching practices that support autonomy, structure, and relatedness are likely to be reflected not only in higher evaluations (Beran & Violato, 2009) but also in healthier motivational and emotional configurations.

An additional contextual consideration concerns the Western Romanian setting in which the study was conducted. Although this regional focus may limit direct generalization to other cultural or institutional contexts, it may also represent a meaningful strength of the study. The sample reflects a higher-education environment shaped by post-transition social, educational, and occupational dynamics, including the coexistence of traditional-age students and adult learners returning to education. From this perspective, the observed network may capture not only individual psychological functioning but also the way academic motivation, self-regulation, and strain are embedded in a regionally specific educational ecology.

Several limitations must be acknowledged. First, the cross-sectional design precludes causal or temporal conclusions. The estimated network represents conditional associations among variables measured at the same time point and, therefore, should not be interpreted as evidence of directional pathways or sequential processes. Longitudinal or intensive repeated-measures network designs would be needed to examine temporal ordering more directly.

Second, all variables were assessed through self-report instruments, which may have introduced common-method variance, shared response tendencies, and subjective reporting biases. As a result, some associations may partly reflect similarities in measurement format or respondent style rather than exclusively substantive psychological relations.

Third, although the manuscript reports the original sources of the instruments together with Romanian validation or adaptation evidence where available, not all measures included in the study benefit from clearly established Romanian psychometric validation, measurement invariance evidence, or confirmatory factor-analytic support in this specific sample. For some instruments, local cultural and linguistic appropriateness remains supported primarily by internal consistency indices rather than by full validation evidence. Accordingly, the findings should be interpreted with caution until broader Romanian validation studies and, where appropriate, sample-specific CFA evidence are available.

Fourth, the interpretation of centrality indices should be undertaken cautiously. Although centrality estimates were useful for describing the relative structural prominence of nodes within the present network, such indices may be sensitive to sampling variation and network estimation choices. Their interpretive value is stronger when accompanied by formal stability evidence; therefore, the centrality findings reported here should be understood as informative but not definitive indicators of node importance.

Fifth, some motivational subscales included in the network are conceptually adjacent and empirically related, particularly within the intrinsic and extrinsic motivation domains. This raises the possibility of partial redundancy or construct overlap, which may inflate associations among closely related nodes and contribute to local clustering. Future studies could address this issue by testing alternative node specifications, latent-variable-informed network models, or redundancy diagnostics prior to network estimation.

Another limitation concerns the multi-university composition of the sample. Participants were recruited from several universities in Western Romania and were analysed as a single pooled sample. Because institutional affiliation was not modelled explicitly, potential between-university differences in academic climate, instructional practices, or student composition could not be examined. Accordingly, some associations observed in the network may partly reflect unmodelled clustering effects at the institutional level.

A further limitation concerns the heterogeneity of participants’ educational level. The sample was not restricted to undergraduate students but included respondents with varying levels of prior and current academic experience, ranging from secondary education to doctoral studies. Because educational level was not modelled as a covariate or stratifying factor, it remains possible that some associations in the network were influenced by differences in academic seniority, prior educational attainment, or stage of study.

Finally, the sample was drawn through convenience procedures from universities in Western Romania and was predominantly female. Although this composition may limit the generalizability of the findings to other student populations, institutional contexts, or gender distributions, it may also be seen as a contextual strength insofar as it captures a regionally specific academic ecology characterized by heterogeneous educational and occupational trajectories.

These findings should be interpreted as describing a conditional association structure rather than a temporally ordered model of academic functioning. Because the present analysis focused on the systemic position of overall burnout rather than on the internal differentiation of its components, the composite score was retained as the most parsimonious representation of burnout in the network.

## 5. Conclusions

Using a network analytic approach, this study indicates that academic burnout occupies a central position in the psychological architecture of students’ academic lives, being closely connected with amotivation, lower self-esteem, higher anxiety, lower engagement, and less favorable perceptions of instruction. In contrast, intrinsic motivation toward achievement, identified regulation, and self-regulated learning processes, especially performance control and forethought, form an adaptive motivational–regulatory core that supports engagement and buffers against burnout.

Rather than viewing burnout, anxiety, motivation, and SRL as separate predictors and outcomes, the network perspective reveals them as interdependent components of a single dynamic system. By identifying which nodes are structurally central and which occupy more intermediary or peripheral positions, the present findings offer a nuanced map of potential leverage points for intervention. Strengthening self-determined forms of motivation and self-regulated learning, while actively monitoring and reducing academic burnout, appears crucial for promoting sustainable engagement and psychological wellbeing among university students.

## Figures and Tables

**Table 1:** Descriptive characteristics of the sample (N = 530).

Variable	Category/Statistic	n	%
Gender	Female	464	87.5
	Male	66	12.5
Educational level	Secondary education	218	41.1
	Post-secondary/vocational	17	3.2
	Bachelor’s degree	206	38.9
	Master’s degree	82	15.5
	Doctoral studies	4	0.8
	Other studies	3	0.6
Marital status	Unmarried	227	42.8
	Married	200	37.7
	In a stable relationship	82	15.5
	Divorced	20	3.8
	Widowed	1	0.2
Occupational status	Public sector employee	204	38.5
	Private sector employee	118	22.3
	Entrepreneur/business owner	21	4.0
	Freelancer	11	2.1
	Not employed (incl. students)	176	33.2
Work experience	<1 year	192	36.2
	1–5 years	137	25.8
	6–10 years	67	12.6
	11–20 years	79	14.9
	>20 years	55	10.4

Age statistics computed on valid cases (n = 527).

**Table 2:** Centrality measures per variable.

Variable	Network			
	Betweenness	Closeness	Strength	Expected Influence
Intrinsic motivation to know	1.180	1.051	0.843	0.515
Intrinsic motivation toward achievement	1.720	1.048	0.911	1.104
Intrinsic motivation to experience stimulation	−0.982	0.383	−0.335	0.373
Extrinsic motivation identified regulation	0.369	0.154	1.057	1.148
Extrinsic motivation introjected regulation	−0.576	−0.442	−0.500	0.374
Extrinsic motivation external regulation	−0.712	−0.729	−0.262	0.170
Amotivation	−0.576	0.679	0.337	−1.053
Generalized anxiety	−0.982	−0.824	−1.854	−0.726
Academic engagement	−0.171	1.089	−0.101	−0.380
Academic burnout	1.991	1.740	1.878	−2.317
Forethought	−0.982	−1.587	−0.350	0.637
Performance control	0.234	−1.010	0.954	1.143
Self-reflection	0.640	−1.038	−0.307	0.472
Self-esteem	−0.171	0.376	−0.898	−1.338
Student ratings of instruction	−0.982	−0.889	−1.374	−0.122

Note: Centrality indices are reported as standardized scores (z-scores) for comparative interpretation across nodes. Therefore, negative values indicate below-average centrality relative to the network mean and do not represent impossible raw values.

**Table 3:** Clustering measures per variable.

Variable	Network			
	Barrat	Onnela	WS	Zhang
Forethought	−0.933	−0.134	−1.370	2.377
Performance control	0.019	−0.268	−0.021	0.375
Self-reflection	0.060	−0.803	0.322	1.857
Self-esteem	−0.592	−0.458	−0.707	−0.297
Universal student ratings of instruction	0.825	−0.347	0.551	0.293
Intrinsic motivation to know	1.521	1.863	1.694	−0.358
Intrinsic motivation toward achievement	−0.371	−0.332	0.665	−0.624
Intrinsic motivation to experience stimulation	1.553	2.240	1.465	0.627
Extrinsic motivation identified regulation	−0.570	−0.769	−0.439	−0.975
Extrinsic motivation introjected regulation	−2.406	−1.484	−1.850	−1.013
Extrinsic motivation external regulation	0.599	0.622	1.008	−0.698
Amotivation	0.628	0.647	−0.090	−0.153
Generalized Anxiety Disorder	−0.565	−0.395	−1.050	0.097
Academic engagement	0.032	−0.696	−0.090	−0.604
Academic burnout	0.199	0.314	−0.090	−0.903

Note: Clustering coefficients are reported as standardized scores (z-scores) for comparative interpretation across nodes. Accordingly, negative values indicate below-average local clustering relative to the network mean rather than negative raw clustering coefficients.

**Table 4:** Weights matrix.

Variable	Network														
	1	2	3	4	5	6	7	8	9	10	11	12	13	14	15
1. IMK	0.000	0.329	0.171	0.348	0.000	−0.050	0.121	0.000	0.006	−0.136	0.000	0.000	0.000	0.000	0.051
2. IMA	0.329	0.000	0.302	0.005	0.347	0.000	0.000	0.000	0.147	0.000	0.000	0.002	0.087	0.000	−0.007
3. IME	0.171	0.302	0.000	0.038	0.107	0.000	−0.100	0.000	0.202	0.000	0.000	0.000	0.000	0.000	0.030
4. EMId	0.348	0.005	0.038	0.000	0.000	0.506	0.129	0.037	0.043	−0.010	0.000	0.021	0.065	0.000	0.058
5. EMIn	0.000	0.347	0.107	0.000	0.000	0.235	−0.051	0.041	0.000	0.016	0.046	0.041	0.000	−0.031	0.000
6. EME	−0.050	0.000	0.000	0.506	0.235	0.000	−0.085	0.000	−0.022	0.053	0.000	0.000	0.000	−0.016	0.000
7. AM	0.121	0.000	−0.100	0.129	−0.051	−0.085	0.000	0.000	−0.063	−0.312	−0.015	0.000	0.000	0.046	0.179
8. GAD	0.000	0.000	0.000	0.037	0.041	0.000	0.000	0.000	0.000	0.178	0.079	0.000	0.000	−0.278	0.000
9. AE	0.006	0.147	0.202	0.043	0.000	−0.022	−0.063	0.000	0.000	−0.279	0.022	0.079	0.004	0.000	0.136
10. AB	−0.136	0.000	0.000	−0.010	0.016	0.053	−0.312	0.178	−0.279	0.000	0.000	0.000	−0.072	−0.253	−0.133
11. FT	0.000	0.000	0.000	0.000	0.046	0.000	−0.015	0.079	0.022	0.000	0.000	0.489	0.296	0.000	0.000
12. PC	0.000	0.002	0.000	0.021	0.041	0.000	0.000	0.000	0.079	0.000	0.489	0.000	0.403	0.128	0.075
13. S-R	0.000	0.087	0.000	0.065	0.000	0.000	0.000	0.000	0.004	−0.072	0.296	0.403	0.000	0.026	0.003
14. S-E	0.000	0.000	0.000	0.000	−0.031	−0.016	0.046	−0.278	0.000	−0.253	0.000	0.128	0.026	0.000	0.047
15. USR	0.051	−0.007	0.030	0.058	0.000	0.000	0.179	0.000	0.136	−0.133	0.000	0.075	0.003	0.047	0.000

Note: IMK = Intrinsic motivation to know; IMA = Intrinsic motivation toward achievement; IME = Intrinsic motivation to experience stimulation; EMId = Identified regulation; EMIn = Introjected regulation; EME = External regulation; AM = Amotivation; GAD = Generalized anxiety; AE = Academic engagement; AB = Academic burnout; FT = Forethought; PC = Performance control; S-R = Self-reflection; S-E = Self-esteem; USR = Student ratings of instruction.

## Data Availability

The dataset generated and analyzed during the current research is available upon reasonable request from the corresponding authors.
